# Effect of type of vaginal preparation on abdominal hysterectomy surgical site infections (SSI)

**DOI:** 10.1017/ice.2025.10258

**Published:** 2025-10

**Authors:** Anupama Neelakanta, Brittany Lees, Tsai-Wei Wang, Kristin Fischer, Catherine Passaretti

**Affiliations:** 1 Section of Infectious Disease, department of Internal Medicine, Wake Forest University School of Medicine, Winston-Salem, NC, USA; 2 Department of Infection Prevention, Advocate Health, Charlotte, NC, USA; 3 Department of Obstetrics and Gynecology, Advocate Health, Charlotte, NC, USA; 4 Department of Clinical Quality Analytics, Advocate Health, Charlotte, NC, USA; 5 Department of Operations and Business Support, Central Division, Advocate Health, Charlotte, NC, USA

## Introduction

Surgical site infections (SSIs), following abdominal hysterectomies, are associated with increased mortality, morbidity, higher medical costs, and increased length of stay. Hysterectomy SSI can significantly affect patient outcomes and also have reputational and financial implications for hospitals. Since these rates are publicly reported and linked to reimbursement, falling below benchmark standards can be particularly consequential.

Hysterectomy SSIs are preventable but require complex interventions. American College of Obstetricians and Gynecologists, Society for Healthcare Epidemiology of America, and Infectious Disease Society of America endorse use of vaginal preparation as part of hysterectomy SSI prevention. While povidone-iodine (PI) is the only Food and Drug Administration approved vaginal preparation agent, SSI prevention guidance from above societies includes recommendations to use either a chlorhexidine (CHG) agent or PI-containing agent for vaginal preparation immediately prior to surgery.^1,2^

While vaginal antisepsis is now standard of care, debate continues regarding the optimal selection of agent for vaginal antisepsis. This study aims to evaluate the impact of CHG compared to PI vaginal preparation on SSI rates following abdominal hysterectomy.

## Methods

We conducted a retrospective study of patients who underwent hysterectomy procedures between January 1, 2020, and January 31, 2024 in a large 10-hospital system (approximately with 2600 beds). We excluded patients with infection present on admission, incomplete data or no vaginal preparation documented. A hysterectomy bundle was introduced in January 2020 which included preoperative CHG bathing, alcohol containing skin preparation, vaginal preparation with CHG or PI per surgeon preference, preoperative intravenous antibiotics per institutional guidelines and, for open cases, the use of a separate closing tray, regowning/regloving, and redraping. Bundle elements were tracked as documented in the EMR. Documentation of closing bundle elements for open cases was poor precluding inclusion in the analysis.

In April 2022, due to persistent hysterectomy SSI rates above benchmark and emerging literature suggesting superior efficacy of CHG for vaginal preparation, the bundle was modified to include CHG as the preferred vaginal preparation agent.^3,4^ Data on compliance with CHG vaginal preparation was monitored and feedback to provider and site-specific operative leadership was given.

Patients who received vaginal preparation with CHG were compared to those who received PI. Categorical demographic and clinical variables were compared using Chi-square tests, while continuous variables were analyzed using t-tests or Mann–Whitney U tests, based on data distribution.

The outcome of interest was all SSIs. SSIs were identified by trained infection preventionists using standard National Healthcare Safety Network (NHSN) definitions.^5^ Patient level demographic and clinical data were extracted from the electronic health record and merged with SSI outcome data at the patient level. Variables included in the multivariate model evaluating impact of vaginal preparation agent on SSI were selected based on significance in both 1) univariate analysis of patients with and without SSI (Supplementary Table 1) and 2) univariate analysis of patients with and without CHG vaginal preparation (Table 1), and 3) established risk factors from prior literature. Age, facility, race, procedure duration, diabetes status, and intraoperative blood loss were included in the final model. A subgroup analysis was conducted in patients who underwent laparoscopic versus open hysterectomy. All analyses were conducted using SPSS software version 29.0.2.0(20) (IBM Corp).

**Table 1.** Demographic and clinical characteristics of patients undergoing abdominal hysterectomy by surgeon choice of vaginal preparation

	All *N* = 10,247		PI *N* = 4,563		CHG *N* = 5,684		*p*-value
Demographics							
Median age, (IQR)	46	(40–54)	46	(40–54)	46	(40–54)	<0.001
Race, *n* (%)							<0.001
White	6,344	(62%)	2,620	(57%)	3,724	(66%)	
Black	2,954	(29%)	1,475	(32%)	1,479	(26%)	
Hispanic	445	(4%)	254	(6%)	191	(3%)	
Other	371	(4%)	182	(4%)	189	(3%)	
Unknown	133	(1%)	32	(1%)	101	(2%)	
Facility, *n* (%)							<0.001
A	1,506	(15%)	84	(2%)	1,422	(25%)	
B	786	(8%)	0	(0%)	786	(14%)	
C	4,603	(45%)	2,500	(55%)	2,103	(37%)	
D	149	(2%)	88	(2%)	61	(1%)	
E	13	(0.1%)	13	(0.3%)	0	(0%)	
F	1,556	(15%)	937	(21%)	619	(11%)	
G	119	(1%)	82	(2%)	37	(1%)	
H	131	(1%)	110	(2%)	21	(0.4%)	
I	80	(1%)	9	(0.2%)	71	(1%)	
J	1,304	(13%)	740	(16%)	564	(10%)	
Clinical Characteristics, n (%)							
Diabetes	1,227	(12%)	537	(12%)	690	(12%)	0.57
Smoking	643	(6%)	168	(4%)	475	(8%)	<0.001
Anemia	4,616	(45%)	2,048	(45%)	2,568	(45%)	0.76
Gynecologic malignancy	1,426	(14%)	694	(15%)	732	(13%)	<0.001
HIV	29	(0.3%)	16	(0.4%)	13	(0.2%)	0.16
Fibroids	6,693	(65%)	3,053	(67%)	3,640	(64%)	0.002
Uterine size >250 grams	2,362	(23%)	1,107	(24%)	1,255	(22%)	<0.001
BMI > 30	5,474	(53%)	2,409	(53%)	3,065	(54%)	0.26
Procedure characteristics							
Median procedure duration, minutes (IQR)	109	(83–145)	111	(86–145)	107	(81–145)	0.004
ASA							0.63
1	467	(5%)	206	(5%)	261	(5%)	
2	6,333	(62%)	2,796	(61%)	3,537	(62%)	
3	3,343	(33%)	1,520	(33%)	1,823	(32%)	
4+	103	(1%)	41	(1%)	62	(1%)	
Wound Class							<0.001
0	1,019	(10%)	182	(4%)	837	(15%)	
1	9,146	(89%)	4,344	(95%)	4,802	(85%)	
2	76	(1%)	35	(1%)	41	(1%)	
3	6	(0.1%)	2	(0%)	4	(0.1%)	
Outpatient procedure	7,202	(70%)	2,950	(65%)	4,252	(75%)	<0.001
Open procedure	1,300	(13%)	722	(16%)	578	(10%)	<0.001
Blood loss > 300 mL	804	(9%)	364	(9%)	440	(8%)	0.04
Chlorhexidine bath given in preoperative area	9,809	(97%)	4,327	(97%)	5,482	(97%)	0.08
Alcohol containing skin preparation	9,356	(92%)	3,953	(88%)	5,403	(95%)	<0.001
Surgical site infections							
SSI	135	(1.3%)	65	(1.4%)	70	(1.2%)	0.4

PI: Povidone iodine; CHG: Chlorhexidine gluconate; HIV: Human immunodeficiency virus; IQR: Interquartile range; BMI: Body mass index

## Results

10,930 patients who underwent abdominal hysterectomies were identified. 683 patients (6.3%) were excluded because they did not have complete data regarding infection prevention process measures (5.1%), were lacking vaginal preparation documentation (1.1%) or had infection present at the time of surgery (0.01%). A total of 10,247 patients who underwent hysterectomies during the study period were included in the analysis. Excluding CHG vaginal preparation, bundle compliance exceeded 90% for elements applicable to all surgeries regardless of subsequent development of an SSI (Supplementary Table 1)

Most procedures (87%) were performed laparoscopically. Baseline demographic and clinical characteristics stratified by vaginal preparation agent are summarized in Table 1. Several differences were found between the CHG and PI groups. Patients who received CHG vaginal preparation were more likely to be White, undergo surgery at certain facilities, be smokers, undergo outpatient surgery, have a clean wound classification and undergo alcohol-based skin preparation. The PI group had higher rates of gynecologic malignancy, fibroids, open procedures, longer procedure duration, and had intraoperative estimated blood loss exceeding 300 ml.

The SSI rate did not differ significantly between vaginal preparation groups in the unadjusted analysis for all procedures (1.4% in PI vs 1.2% in CHG group, *p* = 0.40). Similarly, multivariate logistic regression revealed no significant association between CHG vaginal preparation and overall SSI risk compared to PI (adjusted odds ratio [aOR] 1.05; *p*-value 0.78). In the subgroup analysis for open cases, the adjusted odds ratio for SSI was 0.56 however this did not reach statistical significance (*p* = 0.14) (Table 2).

**Table 2.** Multivariate analysis of risk factors for surgical site infection following abdominal hysterectomy: overall and subgroup analysis by surgical approach

	All *N* = 10247		Laparoscopic (minimally invasive) *N* = 8947		Open *N* = 1300	
	aOR SSI	*p*-value	aOR SSI	*p*-value	aOR SSI	*p*-value
Vaginal preparation with CHG	1.05 (0.7–1.6)	0.78	1.3 (0.8–2.0)	0.335	0.6 (0.3–1.2)	0.14
Age	0.97 (0.95–0.99)	<0.001	0.97 (0.95–0.99)	<0.001	0.97 (0.94–1.01)	0.17
Race/Ethnicity (reference White)		0.9		0.83		0.20
Black	1.1 (0.7–1.6)	0.62	0.9 (0.6–1.5)	0.74	2.1 (0.7–5.7)	0.17
Hispanic	1.3 (0.6–2.7)	0.46	0.6 (0.2–2.0)	0.42	4.3 (1.3–14.3)	0.01
Other	1.0 (0.4–2.6)	0.98	0.6 (0.1–2.4)	0.46	2.9 (0.7–12.3)	0.16
Unknown	0.6 (0.1–4.2)	0.59	0.5 (0.1–4.0)	0.54	0	0.99
Procedure duration (minutes)	1.0 (1.00–1.01)	0.002	1.01 (1.00–1.10)	<0.001	1.01 (0.99–1.01)	0.45
Diabetes	1.6 (1.0–2.7)	0.05	1.7 (0.96–2.9)	0.07	1.5 (0.5–4.1)	0.44
Blood loss >300 ml	2.2 (1.4–3.6)	0.001	1.4 (0.6–2.9)	1.36	3.4 (1.4–7.8)	0.005

aOR: Adjusted odds ratio; CI: 95% Confidence interval.

* The multivariate model was adjusted for CHG vaginal preparation, age, race/ethnicity, facility, procedure duration, diabetes, and intraoperative blood loss > 300 ml. Facility was included in the model but was not significant and thus not shown

## Discussion

Our study aimed to evaluate whether the choice of CHG versus PI for vaginal preparation is associated with the risk of SSI following hysterectomy. Choice of vaginal preparation was not significantly associated with SSI across all procedures. Interestingly, in the subset of open procedures, we found 40% lower adjusted odds of SSI in patients who received CHG vaginal preparation though this did not reach statistical significance. Given that open procedures involve factors that may magnify the importance of effective vaginal antisepsis such as longer operative times and larger incisions, this finding suggests a potential area for future research.

The theoretical advantages of CHG over PI for vaginal preparation are well-established. Studies demonstrate superior vaginal bacterial reduction with CHG compared to PI in addition to CHG’s known bactericidal properties and prolonged antimicrobial activity 4% CHG solution has also shown good tolerability for vaginal preparation.^3,4,6^ Clinical impact on patient outcomes presents a more complex picture. While Lakhi et al found a significant decrease in postcesarean wound infections in patients who received CHG for vaginal preparation compared to PI, studies in hysterectomy patients remain limited.^7^ Available hysterectomy literature to date has shown no significant difference in SSI rates between vaginal preparation agents, which aligns with our findings.^8,9^

While type of vaginal preparation agent did not impact odds of SSI in our analysis, other factors emerged as independent predictors of infection. Younger age, increased procedure duration, diabetes, and intraoperative blood loss greater than 300 mL were associated with higher SSI risk in the overall population. In the subgroup analysis, procedure duration was associated with SSI risk for laparoscopic procedures, while higher intraoperative blood loss was a significant predictor of SSI in open hysterectomies.

Strengths of this study include the large sample size and inclusion of facilities with diverse hysterectomy volumes. However, some limitations should be considered. First, significant baseline demographic and clinical differences existed between patients receiving CHG versus PI. Although we used multivariate modeling to control these differences, residual confounding may persist. Documentation gaps in some of the hysterectomy bundle elements limited the ability to include those factors in the analysis. As this study was conducted within a single health system, findings may have limited generalizability to other populations.

In conclusion, our findings reinforce existing recommendations allowing for either PI or CHG for vaginal preparation prior to hysterectomy. While choice of preparation agent showed minimal overall impact, specific patient populations or procedure types might benefit from one agent over the other. Larger, multicenter, randomized controlled trials are needed to evaluate further.

## Supporting information

Neelakanta et al. supplementary material Neelakanta et al. supplementary material

## Supplementary material

To view supplementary material for this article, please visit https://doi.org/10.1017/ice.2025.10258

## References

[1] Calderwood M , Anderson D , Bratzler D , et al. Strategies to prevent surgical site infections in acute-care hospitals: 2022 Update. Infect Control Hosp Epidemiol 2023;44:695–720 37137483 10.1017/ice.2023.67 PMC10867741

[2] Soper DE , Chelmow D Prevention of infection after gynecologic procedures. Obst & Gynae 2018;131:e172–e189 10.1097/AOG.0000000000002670 29794678

[3] Hill A , Pauls R , Basil J , et al. Chlorhexidine versus iodine for vaginal preparation before hysterectomy: a randomized clinical trial. Female Pelvic Med Reconstr Surg 2022;28: 77–84 34333502 10.1097/SPV.0000000000001066

[4] Culligan P , Kubik K , Murphy M , Blackwell L , Snyder J. A randomized trial that compared povidone iodine and chlorhexidine as antiseptics for vaginal hysterectomy. General Obst and Gynae 2005;192:422–425. 10.1016/j.ajog.2004.08.010 15695981

[5] National Healthcare Safety Network (NHSN) Patient Safety Component Manual. https://www.cdc.gov/nhsn/pdfs/pscmanual/pcsmanual_current.pdf

[6] Dunn D , Yannotti K , Centrella-Nigro A , et al. A Randomized controlled trial of povidone-iodine versus Chlorhexidine gluconate with isopropyl alcohol for preoperative vaginal antisepsis. AORN Journal 2024;119:261–274. 38536409 10.1002/aorn.14111

[7] Lakhi N , Tricorino G , Osipova Y , Moretti M. Vaginal cleansing with chlorhexidine gluconate or povidone-iodine prior to cesarean delivery: A randomized comparator-controlled trial. Am. J. Obstet. Gynecol. MFM 2019;1:2–9. 33319753 10.1016/j.ajogmf.2019.03.004

[8] Duong M , Homewood L. Impact of converting from iodine to Chlorhexidine for vaginal prep prior to hysterectomy. Am J Infect Control 2024;52:87–90. 37595639 10.1016/j.ajic.2023.08.012

[9] Skeith A , Morgan D , Schmidt P. Vaginal preparation with povidone-iodine or chlorhexidine before hysterectomy: A propensity score matched analysis. Am J Obstet Gynecol 2021;225:560.e1–560.e9. 10.1016/j.ajog.2021.08.035 34473965

