# Supplementary material for: Effect of type of vaginal preparation on abdominal hysterectomy surgical site infections (SSI)
Source: Infect Control Hosp Epidemiol. 2025 Sep 16;46(10):1048–51. doi: 10.1017/ice.2025.10258 (PMC12615121; doi:10.1017/ice.2025.10258)
Supplement: Neelakanta et al. supplementary material [file S0899823X25102584sup001.docx]

**Supplemental Table 1: Demographic and Clinical Characteristics of Abdominal Hysterectomy Patients With and Without Surgical Site Infection (SSI).**

|  | **No SSI (%) n = 10315** | **SSI (%) n = 142** | **p-value** |
| --- | --- | --- | --- |
| **Demographics** |  |  |  |
| Mean age in years (SD) | 48 (11.9) | 45 (11.3) | 0.008 |
| Race  White  Black  Hispanic  Other  Unknown | 6,402 (62%) 2,966 (29%) 441 (4%) 372 (4%) 134 (1%) | 70 (49%) 53 (37%) 11 (8%) 6 (4%) 2 (1%) | 0.02 |
| **Procedural characteristics** | | | |
| Emergency | 46 (0.4%) | 3 (2%) | 0.004 |
| Outpatient | 7,221 (70%) | 84 (59%) | 0.005 |
| Laparoscopic | 8,984 (87%) | 104 (73%) | < 0.001 |
| Median Procedure Duration, minutes (IQR) | 109 (83 -145) | 135 (99 -189) | < 0.001 |
| Wound Class  Clean  Clean, Contaminated  Contaminated  Dirty | 1,026 (10%) 9,205 (89%) 78 (1%) 6 (0%) | 15 (11%) 124 (87%) 3 (2%) 0 (0%) | 0.3 |
| ASA  1  2  3  4  5 | 471 (5%) 6,376 (62%) 3,364 (33%) 101 (1%) 2 (0%) | 2 (1%) 74 (52%) 63 (44%) 3 (2%) 0 (0%) | 0.01 |
| **Patient Risk Factors** | | | |
| Smoking | 662 (6%) | 9 (6%) | 1 |
| BMI > 35 | 3,276 (32%) | 65 (46%) | < 0.001 |
| DM | 1,229 (12%) | 27 (19%) | 0.01 |
| Gyn Malignancy | 1,444 (14%) | 20 (14%) | 1 |
| HIV | 31 (0.3%) | 0 (0%) | 0.5 |
| Anemia | 4,629 (45%) | 101 (71%) | < 0.001 |
| Fibroids | 6,727 (65%) | 88 (62%) | 0.4 |
| Blood Loss >300mL | 811 (9%) | 33 (25%) | < 0.001 |
| **Prevention Bundle Elements** | | | |
| Chlorhexidine bath given in preoperative area | 9,871 (96%) | 127 (90%) | < 0.001 |
| Alcohol containing skin prep | 9,386 (92%) | 135 (96%) | 0.2 |
| Vaginal prep completed | 10,199 (99%) | 137 (97%) | 0.008 |
| CHG Vaginal Prep | 5,614 (55%) | 71 (50%) | 0.3 |
